# P-899. Assessing Documented Beta-lactam Allergies at a Large Comprehensive Cancer Hospital

**DOI:** 10.1093/ofid/ofaf695.1106

**Published:** 2026-01-11

**Authors:** Nancy N Vuong, Jovelle Chung, Jianliang Dai, Jovan Borjan, Natalie J Dailey Garnes, Amy Spallone, Guy Handley

**Affiliations:** The University of Texas MD Anderson Cancer Center, Houston, TX; UT MD Anderson, Houston, Texas; UT MD Anderson Cancer Center, Houston, Texas; The University of Texas MD Anderson Cancer Center, Houston, TX; University of Texas MD Anderson Cancer Center, Houston, Texas; University of Texas MD Anderson Cancer Center, Houston, Texas; The University of Texas MD Anderson Cancer Center, Houston, TX

## Abstract

**Background:**

Beta-lactam allergies are documented in up to 10% of the population. True allergic reactions requiring the use of aztreonam, or other alternative antibiotics, account for less than 2% of patients. Avoidance of beta-lactam agents is associated with higher health care costs, increased risk of *Clostridium difficile* infections, and higher mortality rates. We aimed to characterize documented beta-lactam allergies in patients living with cancer.

**Methods:**

This is a single-center retrospective study of hospitalized adult patients living with cancer having a documented beta-lactam allergy between 1/1/2024 through 12/31/2024. We assessed documented reactions, inpatient administration and outpatient prescriptions of antibiotics, and stratified patients for risk of having a true beta-lactam allergy using the PEN-FAST score.

**Results:**

Out of 49,825 hospital admissions, we identified 2658 (5%) patients reporting 2997 beta-lactam allergies. A solid tumor malignancy was the primary cancer diagnosis in 78% of patients. Penicillin (1870/2658, 70%) was the most common documented allergy, followed by a cephalosporin (23%). More than half of patients (57%) had a single reported reaction. Dermatological reactions listed as itching/rash or hives was reported by 49% and 30%, respectively. Anaphylaxis or a severe reaction was reported by 20% of patients. A PEN-FAST score of 0 and 2 was calculated for 779 (29%) and 1470 (55%) patients, respectively. Almost 70% (1831/2658) of patients received at least one dose of a beta-lactam, commonly with a cephalosporin (54%) or a carbapenem (28%), with no documented severe reactions. Aztreonam was administered to 588 (22%) patients in which 82% (484/588) had a PEN-FAST score of 0 or 2. There was no statistical significance in PEN-FAST scores and receiving either aztreonam or an alternative antibiotic.

**Conclusion:**

Evaluation of our patients’ documented beta-lactam allergies revealed that a large portion of patients tolerated a beta-lactam antibiotic. A formal inpatient beta-lactam allergy evaluation service could aid in antimicrobial stewardship efforts to decrease aztreonam use and to remove inpatient beta-lactam allergy labels.

**Disclosures:**

All Authors: No reported disclosures

